# The Effect of LASIK Procedure on Peripapillary Retinal Nerve Fiber Layer and Macular Ganglion Cell-Inner Plexiform Layer Thickness in Myopic Eyes

**DOI:** 10.1155/2017/8923819

**Published:** 2017-09-05

**Authors:** Maja Zivkovic, Vesna Jaksic, Athanassios Giarmoukakis, Michael Grentzelos, Marko Zlatanovic, Gordana Zlatanovic, Aleksandra Miljkovic, Svetlana Jovanovic, Miroslav Stamenkovic, George Kymionis

**Affiliations:** ^1^Medical Faculty, Department of Ophthalmology, University of Nis, Nis, Serbia; ^2^Ophthalmology Clinic, Clinical Center Nis, Nis, Serbia; ^3^Medical Faculty, Department of Ophthalmology, University of Belgrade, Belgrade, Serbia; ^4^Faculty of Medicine, Institute of Vision and Optics (IVO), Vardinoyiannion Eye Institute of Crete, University of Crete, Heraklion, Crete, Greece; ^5^Eye Clinic Maja, Nis, Serbia; ^6^Faculty of Medical Science, Department of Ophthalmology, University of Kragujevac, Kragujevac, Serbia; ^7^FASPER, University of Belgrade, Belgrade, Serbia; ^8^Jules Gonin Eye Hospital, Faculty of Biology and Medicine, University of Lausanne, Lausanne, Switzerland

## Abstract

**Purpose:**

To evaluate the effect of applied suction during microkeratome-assisted laser in situ keratomileusis (LASIK) procedure on peripapillary retinal nerve fiber layer (RNFL) thickness as well as macular ganglion cell-inner plexiform layer (GC-IPL) thickness.

**Methods:**

89 patients (124 eyes) with established myopia range from −3.0 to −8.0 diopters and no associated ocular diseases were included in this study. RNFL and GC-IPL thickness measurements were performed by spectral domain optical coherence tomography (SD OCT) one day before LASIK and at 1 and 6 months postoperatively.

**Results:**

Mean RNFL thickness prior to LASIK was 93.86 ± 12.17 *μ*m while the first month and the sixth month postoperatively were 94.01 ± 12.04 *μ*m and 94.46 ± 12.27 *μ*m, respectively. Comparing results, there is no significant difference between baseline, one month, and six months postoperatively for mean RNFL (*p* > 0.05). Mean GC-IPL thickness was 81.70 ± 7.47 *μ*m preoperatively with no significant difference during the follow-up period (82.03 ± 7.69 *μ*m versus 81.84 ± 7.64 *μ*m; *p* > 0.05).

**Conclusion:**

RNFL and GC-IPL complex thickness remained unaffected following LASIK intervention.

## 1. Introduction

Laser in situ keratomileusis (LASIK) is currently the most common refractive surgery procedure. However, LASIK application among glaucoma patients or glaucoma suspects still remains controversial, mainly because of the short-term increase in intraocular pressure (IOP) during the creation of the corneal flap, that is, during the application of the suction ring. At that point, IOP values may reach up to 65 mmHg [1]. It has been suggested that the transient IOP elevation during LASIK may result in short-term retinal and optic nerve ischemia, which therefore may compromise the structural and functional integrity of these ocular structures [2, 3]. Recent studies, implementing contemporary imaging modalities such as scanning laser polarimetry (SLP) and/or optical coherence tomography (OCT), reported that transient IOP spikes during LASIK have no impact on peripapillary retinal nerve fiber layer (RNFL) thickness [4, 5].

Spectral domain OCT (SD OCT) represents a prevalent imaging technique that among others enables a valid quantitative and qualitative analysis of the peripapillary RNFL [6–9]. Moreover, recent SD OCT software provides a selective evaluation of the inner retinal layers at the macular represent ganglion cell complex (GCC), which includes the nerve fiber layer (NFL), the ganglion cell layer (GCL), and the inner plexiform layer (IPL), providing valuable information regarding early preperimetric glaucomatous ganglion cell damage [10–13]. Furthermore, latest OCT ganglion cell analysis (GCA) algorithms can demarcate the macular ganglion cell-inner plexiform layer (GC-IPL) while excluding the NFL.

Purpose of the present study was to assess the effect of microkeratome-assisted LASIK procedure on the peripapillary RNFL and the GC-IPL using spectral domain OCT (SD OCT).

## 2. Material and Methods

This is a prospective clinic-based observational study that was conducted at the “Maja” Clinic in Nis, Serbia, and was approved by the hospital's ethics committee. All participants were enrolled in the study from the refractive surgery service in a consecutive-if-eligible basis and obtained written informed consent according to the tenets of the Helsinki Declaration.

Inclusion criteria were that participants should have stable refraction for over a year and a spherical equivalent in the range between −3.00 and −8.00 diopters (D) and to be over 18 years old. Exclusion criteria were the presence of any other associated ocular disease, ocular surface disorder, glaucoma, corneal thickness below 500 microns, and irregular corneal topography, as well as any history of systemic disease. Patients who had previous ocular or refractive surgeries were excluded from the study.

All participants received a complete ophthalmological examination, including best-corrected visual acuity, IOP measurement by Goldmann applanation tonometry, gonioscopy, slit lamp and fundus examinations, Schirmer test, corneal pachymetry, and tomography to rule out any LASIK contraindications.

Peripapillary RNFL was measured using the glaucoma analysis mode of Cirrus® SD OCT device (model 4000, software version 6.0, Carl Zeiss Meditec, Inc.). The optic nerve head (ONH) was automatically scanned over an area of 6 × 6 mm by 200 × 200-pixel resolution axial scan. The RNFL thickness within the whole circle circumference, the linear maps in 12 hour positions, and the circular maps in each quadrant was recorded for each patient. The ganglion cell analysis algorithm of the Cirrus SD OCT was used to process and measure the thickness of macular GC-IPL. The average, minimum, and six sectoral (superotemporal, superior, superonasal, inferonasal, inferior, and inferotemporal) GC-IPL thicknesses were measured from the elliptical annulus centered on the fovea. All measurements were performed by an experienced surgeon. Medicament mydriasis was achieved by tropicamide 1% drops before recording. Images with a signal power more than seven were used for analysis. Measurements were performed one day prior to and 1 and 6 months after LASIK.

All LASIK procedures were performed by the same experienced surgeon. Proxymetacaine hydrochloride 0.5% drops were used for local anesthesia, while lids and lashes were sterilized with povidone-iodine (10%) scrub solution. The Moria One Use-Plus SBK microkeratome was used for the creation of the flap. The negative pressure of the suction ring was set at 600–620 mmHg, and the velocity of the head movement was constant (3 mm/seconds). The hinge was created at the 12 o'clock position. The Alcon WaveLight Allegretto Wave Eye-Q® excimer laser 400 Hz was used for all ablations. After ablation, the flap was repositioned with an irrigation cannula and the interface was thoroughly irrigated. In postoperative period, all patients were administered fixed combination of tobramycin and dexamethasone q.i.d. for a 10 days and preservative free artificial tears for 2 months.

### 2.1. Statistical Analysis

Kolmogorov-Smirnov testing was applied to the assessment of the normality of the measured data. All parameters were expressed as mean ± standard deviation (SD). Differences between pre- and postoperative measurements were evaluated by means of Mann–Whitney test. The level of statistical significance was set at *p* < 0.05. All statistical analyses were performed with the Statistical Program for Social Sciences (SPSS) version 20.0 for Windows software package (SPSS, Inc., Chicago, IL, USA).

## 3. Results

In total, 89 patients (124 eyes) were included in the study; 56 were female (62.92%), mean age 32.08 ± 7.7 years (range 18–51 years). The mean preoperative spherical equivalent was −4.81 ± 2.48 D. Preoperative and postoperative RNFL values at all quadrants and in each of the 12 clock-hour sectors (marked as sectors at 1 h, 2 h,…, 12 h, adjusted and aligned for the left and right eye) are shown in Table 1. Mean RNFL thickness prior to LASIK was 93.86 ± 12.17 at the baseline, the first postoperative month was 94.01 ± 12.04, and the sixth postoperative month was 94.46 ± 12.27 *μ*m. Comparing results, there is no significant difference between baseline, one month and six months postoperatively for mean RNFL (*p* > 0.05) as well as regarding the clock hours and quadrants RNFL thickness.

Preoperative and postoperative mean and minimum values of GC-IPL as well as GC-IPL thickness in the superotemporal (ST), superior (S), superonasal (SN), inferonasal (IN), inferior (I), and inferotemporal (IT) layer have been reported in Table 2. The mean preoperative value of GC-IPL thickness was 81.70 ± 7.47 *μ*m while the minimum value of GC-IPL thickness was 75.21 ± 12.57 *μ*m. The GC-IPL complex thickness did not change significantly from preoperative to any postoperative visit (*p* > 0.05). In addition, the mean central subfield thickness was 263.47 ± 21.46, preoperatively, with any significant differences being detected at follow-up visits (Table 2).

## 4. Discussion

A series of previous studies regarding the influence of transient intraoperative IOP elevation during LASIK on RNFL thickness, implementing conventional SLP with fixed compensator, reported a postoperative thinning of RNFL, raising considerations concerning the safety of LASIK in patients with glaucoma or glaucoma suspects. Nevertheless, this finding was attributed to alterations in corneal birefringence following LASIK that affected the accuracy of the instrument's readings [5, 14–17]. This was further supported by similar reports, which using SLP with variable corneal compensator concluded that RNFL actually remained unaffected following LASIK, when compensating individually for changes in corneal birefringence [17, 18]. In addition, more recent studies utilizing OCT analysis found no LASIK induced peripapillary RNFL alterations [4, 5, 15, 16, 19, 20].

A great importance in establishing glaucoma diagnosis and in monitoring structural changes in glaucomatous patients has lately been attributed to GCC [10–12]. GCC is defined as the sum of NFL, ganglion cell layer, and inner plexiform layers at the macular region. Glaucoma likely preferentially affects these layers rather than all macular layers, because they contain the ganglion cell axons, cell bodies, and dendrites [11]. Moreover, studies supported that GCC analysis has similar glaucoma discriminating performance compared with the peripapillary RNFL thickness evaluation [11, 21], while outbalancing RNFL's diagnostic capacity in certain cases [22]. However, it was questioned whether the inclusion of NFL thickness in GCC thickness measurements falsely elevated the diagnostic performance of the GCC. Therefore, the latest GCC analysis algorithms facilitate the successful demarcation of the macular ganglion cell-inner plexiform layer (GC-IPL), while excluding the NFL [21, 23].

In the current study, we attempted to evaluate the effect of applied suction during microkeratome-assisted LASIK on OCT-derived RNFL and macular GC-IPL thickness, in a cohort of myopic patients without any other ocular pathology. According to our findings, suction-induced IOP elevation during LASIK produces nonsignificant alterations on the OCT-derived peripapillary RNFL, which is in agreement with previous literature reports [4, 5, 16, 19, 20]. Zhang et al. [13] reported nonsignificant changes in both RNFL and GCC thickness after femtosecond laser-assisted LASIK and femtosecond lenticule extraction. Our results additionally suggest that GC-IPL thickness, namely, the macular GCC without the NFL thickness, remains unaffected following microkeratome-assisted LASIK, as well.

## 5. Conclusion

In conclusion, to the best of our knowledge, this is one of the first studies to report on the impact of microkeratome-assisted LASIK on peripapillary RNFL, as well as the macular GC-IPL thickness, excluding influence of macular NFL. Our results suggest that suction-induced IOP elevation has no clinically significant impact on RNFL and macular GC-IPL thickness, namely, the retinal structures that are most susceptible to elevated IOP.

## Figures and Tables

**Table 1:** Mean RNFL thickness (*μ*m) and RNFL thickness in 4 quadrants and in 12 sectors measured by OCT before and 1 and 6 months after microkeratome-assisted LASIK.

Parameters	Preoperative (mean ± SD)	1 month postoperatively (mean ± SD)	*p* value	6 months postoperatively (mean ± SD)	*p* value
RNFL Avg	93.86 ± 12.17	94.01 ± 12.04	0.088	94.46 ± 12.27	0.053
RNFL T	62.25 ± 12.09	62.78 ± 12.55	0.138	63.04 ± 12.11	0.245
RNFL N	65.86 ± 11.33	65.69 ± 11.51	0.596	66.07 ± 11.76	0.063
RNFL I	108.58 ± 17.20	108.69 ± 17.23	0.763	109.10 ± 16.88	0.151
RNFL S	101.28 ± 18.57	100.44 ± 18.32	0.051	101.21 ± 18.56	0.861
1 h	90.18 ± 18.24	90.57 ± 18.27	0.069	90.63 ± 18.38	0.054
2 h	84.04 ± 18.77	84.11 ± 18.90	0.844	83.96 ± 19.01	0.869
3 h	55.03 ± 9.93	55.18 ± 10.70	0.063	55.25 ± 10.85	0.056
4 h	58.80 ± 12.59	59.08 ± 13.64	0.433	59.25 ± 12.80	0.063
5 h	84.66 ± 21.94	84.75 ± 22.54	0.794	84.97 ± 21.90	0.391
6 h	119.15 ± 23.92	119.28 ± 23.74	0.692	119.79 ± 24.0	0.063
7 h	120.32 ± 21.87	120.67 ± 21.87	0.063	120.86 ± 22.16	0.052
8 h	62.28 ± 11.36	62.41 ± 11.84	0.454	62.68 ± 11.87	0.272
9 h	52.39 ± 14.96	52.96 ± 13.14	0.496	53.89 ± 13.67	0.057
10 h	72.53 ± 19.67	74.35 ± 18.65	0.106	74.55 ± 17.76	0.076
11 h	114.16 ± 26.22	117.17 ± 23.24	0.116	116.53 ± 23.06	0.226
12 h	102.61 ± 23.74	104.51 ± 20.96	0.212	104.41 ± 21.16	0.240

RNFL Avg, retinal nerve fiber layer average; RNFL T, retinal nerve fiber layer in temporal quadrant; RNFL N, retinal nerve fiber layer in nasal quadrant; RNFL I, retinal nerve fiber layer in inferior quadrant; RNFL S, retinal nerve fiber layer in superior quadrant; 1 h–12 h, retinal nerve fiber layer in 12 RNFL clock hours; SD, standard deviation.

**Table 2:** Mean GC-IPL thickness average, minimum, and in 6 sectors and central subfield thickness in *μ*m measured by OCT before and 1 and 6 months after microkeratome-assisted LASIK.

Parameters	Preoperative (mean ± SD)	1 month postoperatively (mean ± SD)	*p* value	6 months postoperatively (mean ± SD)	*p* value
GC-IPL Avg	81.70 ± 7.47	82.03 ± 7.69	0.193	81.84 ± 7.64	0.579
GC-IPL Min	75.21 ± 12.57	76.20 ± 12.55	0.174	75.26 ± 13.32	0.860
GC-IPL 1	84.19 ± 11.90	84.59 ± 11.85	0.248	84.73 ± 11.87	0.059
GC-IPL 2	80.81 ± 9.85	81.12 ± 10.39	0.347	81.02 ± 9.87	0.295
GC-IPL 3	80.30 ± 9.34	80.07 ± 9.52	0.505	80.45 ± 9.56	0.645
GC-IPL 4	82.71 ± 7.54	83.29 ± 8.22	0.106	82.61 ± 8.26	0.755
GC-IPL 5	80.35 ± 9.98	80.74 ± 10.47	0.163	80.74 ± 10.29	0.316
GC-IPL 6	81.86 ± 10.40	82.00 ± 11.04	0.606	82.06 ± 11.04	0.519
CST	263.47 ± 21.46	259.39 ± 22.32	0.481	259.21 ± 25.46	0.401

GC-IPL, ganglion cell layer + inner plexiform layer; Avg, average; Min, minimum; ST, superotemporal; S, superior; SN, superonasal; IN, inferonasal; I, inferior; IT, inferotemporal; CST, central subfield thickness; SD, standard deviation.
